# Disopropyl {[(2*S*,3*S*)-2-amino-3-methyl­penta­namido](phen­yl)meth­yl}phosphinate

**DOI:** 10.1107/S1600536810051354

**Published:** 2010-12-18

**Authors:** Hong-Ming Cheng, Han-Wen Zhang, Hua Fang, Zhen Wu, Yu-Fen Zhao

**Affiliations:** a Department of Chemistry, Key Laboratory for Chemical Biology of Fujian Province, College of Chemistry and Chemical Engineering, Xiamen University, Xiamen 361005, People’s Republic of China; b The Third Institute of Oceanography of the State Oceanic Administration, Xiamen 361005, People’s Republic of China; c Department of Pharmaceutical Science, Medical College, Xiamen University, Xiamen 361005, People’s Republic of China

## Abstract

There are two independent mol­ecules in the asymmetric unit of the title compound, C_19_H_33_N_2_O_4_P. In the crystal, the two independent mol­ecules are linked *via* N—H⋯O=P hydrogen bonds, forming dimers.

## Related literature

For the biological activity of phosphono-peptides, see: Li *et al.* (1999 ▶); Liu *et al.* (2002 ▶); Wang *et al.* (2001 ▶); Senten *et al.* (2003 ▶); Joossens & Van der Veken (2004 ▶).
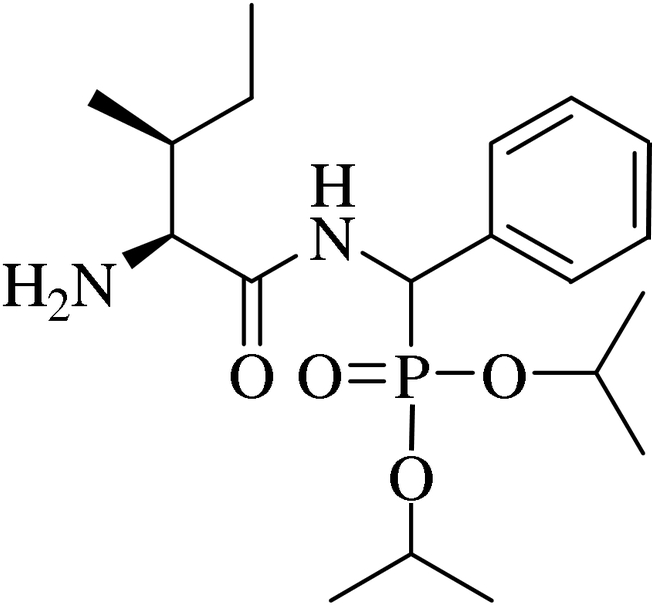


## Experimental

### 

#### Crystal data

C_19_H_33_N_2_O_4_P
*M* *_r_* = 384.44
Monoclinic, 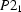

*a* = 9.3455 (3) Å
*b* = 23.6079 (6) Å
*c* = 10.0517 (4) Å
β = 103.819 (4)°
*V* = 2153.49 (12) Å^3^
*Z* = 4
Mo *K*α radiation
μ = 0.15 mm^−1^
*T* = 293 K
0.37 × 0.22 × 0.18 mm

#### Data collection

Bruker APEX area-detector diffractometer
Absorption correction: multi-scan (*SADABS*; Bruker, 2001 ▶) *T*_min_ = 0.946, *T*_max_ = 0.973
7721 measured reflections
6100 independent reflections
4077 reflections with *I* > 2σ(*I*)
*R*_int_ = 0.028

#### Refinement

*R*[*F* ^2^ > 2σ(*F* ^2^)] = 0.043
*wR*(*F* ^2^) = 0.068
*S* = 0.82
6100 reflections
469 parameters
1 restraint
H-atom parameters constrained
Δρ_max_ = 0.25 e Å^−3^
Δρ_min_ = −0.21 e Å^−3^
Absolute structure: Flack (1983 ▶), 2215 Friedel pairs
Flack parameter: −0.04 (8)

### 

Data collection: *SMART* (Bruker, 2007 ▶); cell refinement: *SAINT* (Bruker, 2007 ▶); data reduction: *SAINT*; program(s) used to solve structure: *SHELXS97* (Sheldrick, 2008 ▶); program(s) used to refine structure: *SHELXL97* (Sheldrick, 2008 ▶); molecular graphics: *ORTEPIII* (Farrugia, 1997 ▶) and *PLATON* (Spek, 2009 ▶); software used to prepare material for publication: *SHELXL97*.

## Supplementary Material

Crystal structure: contains datablocks I, global. DOI: 10.1107/S1600536810051354/su2222sup1.cif

Structure factors: contains datablocks I. DOI: 10.1107/S1600536810051354/su2222Isup2.hkl

Additional supplementary materials:  crystallographic information; 3D view; checkCIF report

## Figures and Tables

**Table 1:** Hydrogen-bond geometry (Å, °)

*D*—H⋯*A*	*D*—H	H⋯*A*	*D*⋯*A*	*D*—H⋯*A*
N1—H1*A*⋯O2′^i^	0.86	2.07	2.913 (4)	166
N1′—H1′*A*⋯O2^ii^	0.86	1.98	2.833 (4)	171

Symmetry codes: (i) 
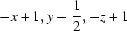
; (ii) 
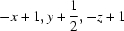
.

**Fig. 1.**
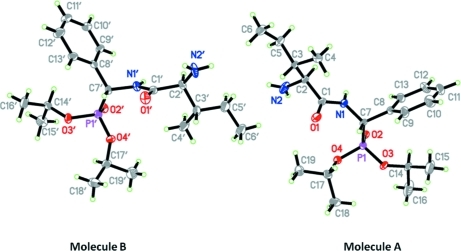
The molecular structure of the two indendent molecules (A and B) of the title compound, with displacement ellipsoids drawn at the 30% probability level. The H-atoms are drawn as spheres of arbitrary radii.

**Fig. 2.**
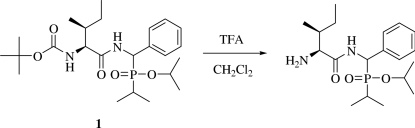
The formation of the title compound.

## supplementary crystallographic information

### Comment

In recent years phosphono-peptides have stimulated a great deal of interest due to their considerable biological activities, including antigrowth (Li *et al.*, 1999), antitumoral (Liu *et al.*, 2002), antiviral (Wang *et al.*, 2001), and inhibiton of serine protease effects (Senten *et al.*, 2003 and Joossens *et al.*, 2004).

The title compound crystallized with two independent chiral molecules (A and B) in the asymmetric unit (Fig. 1). They differ only in the chirality of atom C7 (molecule A) and C7' (molecule B).

In the crystal the two independent molecules are linked via N—H···O=P hydrogen bonds, involving the amide unit and a phosphoryl O atom, to form dimers (Table 1).

### Experimental

To a solution of the starting material, tert-butyl (2S,3S)-1-((isopropoxy(isopropyl)phosphoryl)(phenyl)methylamino) -3-methyl-1-oxopentan-2-ylcarbamate, (1 in scheme), (1 mmol) in dichloromethane (CH_2_Cl_2_) (10 ml) at 273 K was added trifluoroacetic acid (TFA) (4 ml). After consumption of the starting material (2h), the solvent was then removed under reduced pressure to give a residue, which was extracted with CH_2_Cl_2_ (3 × 15 ml). The organic phase was dried over anhydrous MgSO_4_ and concentrated under vacuum to obtain a slurry residue, which was purified by silica gel column chromatography (petroleum ether/isopropyl alcohol = 35:1) to give the title compound as a colorless amorphous solid. Single crystals of the title compound, suitable for X-ray diffraction analysis, were obtained by slow evaporation of a CH_2_Cl_2_ solution.

### Refinement

All H atoms were placed in geometrically idealized positions and treated as riding on their parent atoms, with C—H = 0.93 (aromatic), 0.96 (CH_3_), 0.97 (CH_2_) and 0.98 (CH), N—H = 0.86 Å with *U*_iso_(H) = k × *U*_eq_(C or N), where k = 1.5 for methyl and amine H-atoms and 1.2 for all other H-atoms.

### Crystal data

**Table:**

C_19_H_33_N_2_O_4_P	*F*(000) = 832
*M**_r_* = 384.44	*D*_x_ = 1.186 Mg m^−^^3^
Monoclinic, *P*2_1_	Mo *K*α radiation, λ = 0.71073 Å
Hall symbol: P 2yb	Cell parameters from 2328 reflections
*a* = 9.3455 (3) Å	θ = 2.2–27.4°
*b* = 23.6079 (6) Å	µ = 0.15 mm^−^^1^
*c* = 10.0517 (4) Å	*T* = 293 K
β = 103.819 (4)°	Block, colourless
*V* = 2153.49 (12) Å^3^	0.37 × 0.22 × 0.18 mm
*Z* = 4	

### Data collection

**Table:**

Bruker APEX area-detector diffractometer	6100 independent reflections
Radiation source: fine-focus sealed tube	4077 reflections with *I* > 2σ(*I*)
graphite	*R*_int_ = 0.028
φ and ω scans	θ_max_ = 25.0°, θ_min_ = 2.2°
Absorption correction: multi-scan (*SADABS*; Bruker, 2001)	*h* = −11→10
*T*_min_ = 0.946, *T*_max_ = 0.973	*k* = −28→27
7721 measured reflections	*l* = −10→11

### Refinement

**Table:**

Refinement on *F*^2^	Secondary atom site location: difference Fourier map
Least-squares matrix: full	Hydrogen site location: inferred from neighbouring sites
*R*[*F*^2^ > 2σ(*F*^2^)] = 0.043	H-atom parameters constrained
*wR*(*F*^2^) = 0.068	*w* = 1/[σ^2^(*F*_o_^2^) + (0.0212*P*)^2^] where *P* = (*F*_o_^2^ + 2*F*_c_^2^)/3
*S* = 0.82	(Δ/σ)_max_ < 0.001
6100 reflections	Δρ_max_ = 0.25 e Å^−^^3^
469 parameters	Δρ_min_ = −0.21 e Å^−^^3^
1 restraint	Absolute structure: Flack (1983), 2215 Friedel pairs
Primary atom site location: structure-invariant direct methods	Flack parameter: −0.04 (8)

### Special details

**Table:**

Geometry. All e.s.d.'s (except the e.s.d. in the dihedral angle between two l.s. planes) are estimated using the full covariance matrix. The cell e.s.d.'s are taken into account individually in the estimation of e.s.d.'s in distances, angles and torsion angles; correlations between e.s.d.'s in cell parameters are only used when they are defined by crystal symmetry. An approximate (isotropic) treatment of cell e.s.d.'s is used for estimating e.s.d.'s involving l.s. planes.
Refinement. Refinement of *F*^2^ against ALL reflections. The weighted *R*-factor *wR* and goodness of fit *S* are based on *F*^2^, conventional *R*-factors *R* are based on *F*, with *F* set to zero for negative *F*^2^. The threshold expression of *F*^2^ > σ(*F*^2^) is used only for calculating *R*-factors(gt) *etc*. and is not relevant to the choice of reflections for refinement. *R*-factors based on *F*^2^ are statistically about twice as large as those based on *F*, and *R*- factors based on ALL data will be even larger.

### Fractional atomic coordinates and isotropic or equivalent isotropic displacement parameters (Å^2^)

**Table:**

	*x*	*y*	*z*	*U*_iso_*/*U*_eq_	
P1	0.54488 (9)	0.12742 (4)	0.41034 (10)	0.0250 (2)	
P1'	0.76424 (10)	0.71698 (4)	0.26570 (11)	0.0290 (2)	
O3	0.5955 (2)	0.07253 (9)	0.3476 (3)	0.0301 (6)	
O3'	0.8151 (2)	0.77426 (9)	0.2128 (3)	0.0332 (6)	
O2	0.5256 (2)	0.12060 (9)	0.5501 (2)	0.0283 (6)	
O2'	0.7782 (2)	0.71540 (10)	0.4143 (2)	0.0348 (6)	
N1	0.3215 (3)	0.20195 (10)	0.3342 (3)	0.0265 (7)	
H1A	0.2822	0.2011	0.4033	0.032*	
O4	0.6659 (2)	0.17261 (9)	0.4084 (2)	0.0284 (6)	
O1	0.3933 (3)	0.25766 (9)	0.1783 (3)	0.0411 (7)	
O4'	0.8531 (2)	0.67185 (9)	0.2055 (3)	0.0318 (6)	
N1'	0.5259 (3)	0.64986 (10)	0.1920 (3)	0.0285 (8)	
H1'A	0.5203	0.6421	0.2742	0.034*	
C7'	0.5793 (3)	0.70512 (12)	0.1630 (4)	0.0266 (9)	
H7'A	0.5834	0.7056	0.0665	0.032*	
C1'	0.4849 (4)	0.61045 (15)	0.0934 (4)	0.0314 (9)	
C7	0.3786 (3)	0.14968 (12)	0.2894 (4)	0.0234 (9)	
H7A	0.4048	0.1578	0.2026	0.028*	
O1'	0.4953 (3)	0.61820 (11)	−0.0246 (3)	0.0452 (7)	
C8'	0.4764 (3)	0.75257 (14)	0.1842 (4)	0.0253 (9)	
C14'	0.8065 (4)	0.82932 (15)	0.2768 (4)	0.0371 (10)	
H14A	0.7221	0.8299	0.3189	0.045*	
C8	0.2625 (4)	0.10308 (13)	0.2619 (4)	0.0261 (9)	
C2	0.2534 (4)	0.30147 (13)	0.3222 (4)	0.0336 (10)	
H2A	0.1866	0.2859	0.3748	0.040*	
C3	0.1619 (3)	0.33578 (13)	0.2048 (4)	0.0277 (9)	
H3A	0.2298	0.3545	0.1581	0.033*	
C3'	0.5447 (4)	0.50929 (14)	0.1574 (4)	0.0384 (11)	
H3'A	0.5721	0.5035	0.0702	0.046*	
C1	0.3288 (4)	0.25194 (15)	0.2697 (4)	0.0298 (9)	
C9'	0.4235 (4)	0.75559 (14)	0.3020 (4)	0.0341 (10)	
H9'A	0.4504	0.7280	0.3694	0.041*	
C2'	0.4246 (4)	0.55510 (14)	0.1336 (4)	0.0342 (10)	
H2'A	0.3939	0.5609	0.2193	0.041*	
C14	0.5785 (4)	0.01592 (14)	0.3957 (5)	0.0405 (11)	
H14B	0.5159	0.0171	0.4612	0.049*	
C9	0.2107 (4)	0.08209 (15)	0.1318 (5)	0.0458 (11)	
H9A	0.2456	0.0966	0.0595	0.055*	
C12'	0.3435 (4)	0.83763 (18)	0.1064 (5)	0.0591 (13)	
H12A	0.3152	0.8653	0.0394	0.071*	
N2	0.3607 (3)	0.33692 (12)	0.4136 (4)	0.0514 (9)	
H2B	0.3655	0.3725	0.3969	0.062*	
H2C	0.4194	0.3223	0.4844	0.062*	
C5	0.0744 (4)	0.38135 (14)	0.2554 (4)	0.0449 (11)	
H5A	0.1355	0.3985	0.3372	0.054*	
H5B	−0.0098	0.3641	0.2804	0.054*	
N2'	0.2973 (3)	0.53769 (12)	0.0285 (4)	0.0572 (11)	
H2'B	0.2672	0.5582	−0.0435	0.069*	
H2'C	0.2519	0.5068	0.0380	0.069*	
C5'	0.4878 (5)	0.45359 (15)	0.1966 (5)	0.0617 (14)	
H5'A	0.4002	0.4435	0.1272	0.074*	
H5'B	0.4588	0.4586	0.2822	0.074*	
C10'	0.3322 (4)	0.79868 (15)	0.3199 (4)	0.0397 (10)	
H10A	0.2963	0.7999	0.3986	0.048*	
C6	0.0196 (4)	0.42788 (15)	0.1484 (5)	0.0558 (13)	
H6A	−0.0365	0.4553	0.1851	0.084*	
H6B	−0.0414	0.4113	0.0673	0.084*	
H6C	0.1025	0.4462	0.1260	0.084*	
C17	0.7184 (4)	0.19043 (14)	0.2890 (4)	0.0378 (10)	
H17A	0.6411	0.1839	0.2055	0.045*	
C13'	0.4373 (4)	0.79381 (15)	0.0870 (4)	0.0392 (10)	
H13A	0.4729	0.7928	0.0081	0.047*	
C4	0.0620 (4)	0.29675 (15)	0.1011 (4)	0.0440 (11)	
H4A	0.1211	0.2692	0.0687	0.066*	
H4B	0.0081	0.3188	0.0253	0.066*	
H4C	−0.0058	0.2778	0.1443	0.066*	
C13	0.2073 (4)	0.08177 (15)	0.3678 (4)	0.0391 (11)	
H13B	0.2390	0.0966	0.4557	0.047*	
C18'	1.0836 (4)	0.67632 (19)	0.1413 (5)	0.0633 (14)	
H18A	1.0643	0.7143	0.1077	0.095*	
H18B	1.0433	0.6500	0.0694	0.095*	
H18C	1.1881	0.6706	0.1715	0.095*	
C11'	0.2934 (4)	0.83991 (17)	0.2233 (5)	0.0507 (12)	
H11A	0.2329	0.8696	0.2368	0.061*	
C17'	1.0145 (4)	0.66720 (16)	0.2576 (4)	0.0400 (10)	
H17B	1.0494	0.6959	0.3284	0.048*	
C11	0.0566 (4)	0.01705 (18)	0.2126 (6)	0.0605 (14)	
H11B	−0.0106	−0.0127	0.1958	0.073*	
C16	0.7231 (5)	−0.00693 (16)	0.4637 (6)	0.090 (2)	
H16A	0.7652	0.0155	0.5430	0.136*	
H16B	0.7867	−0.0060	0.4015	0.136*	
H16C	0.7123	−0.0453	0.4911	0.136*	
C15	0.5065 (5)	−0.02030 (18)	0.2762 (6)	0.094 (2)	
H15A	0.4094	−0.0061	0.2366	0.141*	
H15B	0.4999	−0.0586	0.3065	0.141*	
H15C	0.5641	−0.0194	0.2089	0.141*	
C6'	0.5957 (5)	0.40500 (16)	0.2128 (6)	0.097 (2)	
H6'A	0.5496	0.3711	0.2348	0.145*	
H6'B	0.6805	0.4134	0.2851	0.145*	
H6'C	0.6256	0.3996	0.1287	0.145*	
C19	0.7503 (4)	0.25248 (15)	0.3056 (5)	0.0517 (12)	
H19A	0.6606	0.2727	0.3031	0.078*	
H19B	0.7920	0.2655	0.2325	0.078*	
H19C	0.8189	0.2591	0.3918	0.078*	
C15'	0.9436 (4)	0.83977 (18)	0.3822 (4)	0.0576 (13)	
H15D	0.9513	0.8130	0.4554	0.086*	
H15E	0.9421	0.8775	0.4174	0.086*	
H15F	1.0267	0.8356	0.3423	0.086*	
C19'	1.0489 (4)	0.60907 (16)	0.3168 (5)	0.0689 (15)	
H19D	1.0076	0.6047	0.3950	0.103*	
H19E	1.1538	0.6041	0.3442	0.103*	
H19F	1.0072	0.5812	0.2490	0.103*	
C16'	0.7826 (5)	0.87172 (15)	0.1608 (5)	0.0604 (14)	
H16D	0.6898	0.8644	0.0978	0.091*	
H16E	0.8605	0.8684	0.1141	0.091*	
H16F	0.7823	0.9093	0.1971	0.091*	
C12	0.1044 (4)	0.03818 (17)	0.3426 (5)	0.0515 (12)	
H12B	0.0680	0.0234	0.4137	0.062*	
C10	0.1072 (5)	0.03950 (19)	0.1086 (5)	0.0602 (13)	
H10B	0.0715	0.0259	0.0200	0.072*	
C4'	0.6813 (4)	0.52743 (15)	0.2605 (5)	0.0594 (14)	
H4'A	0.7552	0.4985	0.2690	0.089*	
H4'B	0.6585	0.5333	0.3476	0.089*	
H4'C	0.7177	0.5620	0.2306	0.089*	
C18	0.8524 (4)	0.15616 (16)	0.2835 (5)	0.0625 (15)	
H18D	0.8254	0.1170	0.2686	0.094*	
H18E	0.9249	0.1600	0.3685	0.094*	
H18F	0.8925	0.1696	0.2098	0.094*	

### Atomic displacement parameters (Å^2^)

**Table:**

	*U*^11^	*U*^22^	*U*^33^	*U*^12^	*U*^13^	*U*^23^
P1	0.0293 (5)	0.0232 (5)	0.0231 (6)	0.0013 (4)	0.0075 (5)	−0.0012 (5)
P1'	0.0313 (5)	0.0299 (6)	0.0274 (7)	−0.0014 (5)	0.0100 (5)	0.0004 (5)
O3	0.0392 (13)	0.0222 (13)	0.0314 (17)	0.0077 (11)	0.0134 (13)	0.0018 (12)
O3'	0.0485 (15)	0.0234 (14)	0.0322 (17)	−0.0054 (12)	0.0186 (14)	−0.0032 (13)
O2	0.0369 (13)	0.0296 (14)	0.0209 (15)	0.0029 (11)	0.0117 (12)	−0.0018 (13)
O2'	0.0434 (14)	0.0386 (14)	0.0243 (15)	−0.0068 (13)	0.0114 (13)	0.0001 (14)
N1	0.0382 (17)	0.0211 (17)	0.0247 (19)	0.0043 (13)	0.0167 (16)	0.0048 (15)
O4	0.0338 (13)	0.0297 (13)	0.0244 (16)	−0.0032 (11)	0.0121 (13)	−0.0035 (12)
O1	0.0585 (17)	0.0320 (16)	0.043 (2)	0.0109 (13)	0.0331 (16)	0.0119 (14)
O4'	0.0282 (13)	0.0339 (14)	0.0335 (17)	0.0039 (11)	0.0074 (13)	−0.0028 (13)
N1'	0.0366 (16)	0.0302 (18)	0.0180 (19)	−0.0070 (13)	0.0049 (16)	0.0008 (15)
C7'	0.0359 (19)	0.025 (2)	0.022 (2)	−0.0058 (17)	0.0134 (18)	0.0030 (18)
C1'	0.030 (2)	0.034 (2)	0.031 (3)	0.0018 (17)	0.009 (2)	−0.002 (2)
C7	0.0307 (19)	0.023 (2)	0.018 (2)	0.0045 (15)	0.0107 (18)	−0.0022 (17)
O1'	0.0679 (18)	0.0463 (17)	0.0259 (17)	−0.0096 (14)	0.0199 (15)	−0.0034 (15)
C8'	0.0240 (19)	0.028 (2)	0.024 (3)	−0.0037 (16)	0.0054 (19)	0.0019 (19)
C14'	0.038 (2)	0.031 (2)	0.047 (3)	−0.0045 (18)	0.019 (2)	−0.005 (2)
C8	0.0291 (19)	0.023 (2)	0.026 (2)	0.0018 (16)	0.0051 (19)	0.0037 (18)
C2	0.039 (2)	0.023 (2)	0.043 (3)	0.0058 (17)	0.020 (2)	0.001 (2)
C3	0.034 (2)	0.0202 (19)	0.033 (3)	0.0032 (17)	0.0156 (19)	0.0034 (18)
C3'	0.052 (2)	0.029 (2)	0.039 (3)	−0.0053 (19)	0.023 (2)	−0.004 (2)
C1	0.033 (2)	0.027 (2)	0.033 (3)	0.0044 (18)	0.016 (2)	0.001 (2)
C9'	0.036 (2)	0.031 (2)	0.036 (3)	0.0073 (18)	0.010 (2)	0.006 (2)
C2'	0.037 (2)	0.032 (2)	0.036 (3)	−0.0107 (18)	0.015 (2)	−0.002 (2)
C14	0.058 (3)	0.019 (2)	0.057 (3)	0.002 (2)	0.038 (3)	0.003 (2)
C9	0.050 (2)	0.041 (2)	0.044 (3)	−0.008 (2)	0.006 (2)	−0.013 (2)
C12'	0.063 (3)	0.058 (3)	0.064 (4)	0.025 (2)	0.030 (3)	0.028 (3)
N2	0.065 (2)	0.0335 (19)	0.045 (3)	0.0142 (17)	−0.0096 (19)	−0.0124 (18)
C5	0.056 (2)	0.030 (2)	0.052 (3)	0.0140 (19)	0.018 (2)	0.004 (2)
N2'	0.059 (2)	0.040 (2)	0.061 (3)	−0.0297 (17)	−0.010 (2)	0.007 (2)
C5'	0.076 (3)	0.039 (3)	0.080 (4)	0.002 (2)	0.038 (3)	0.002 (3)
C10'	0.035 (2)	0.045 (2)	0.040 (3)	0.0044 (19)	0.012 (2)	−0.003 (2)
C6	0.055 (3)	0.039 (3)	0.075 (4)	0.018 (2)	0.020 (3)	0.016 (3)
C17	0.040 (2)	0.043 (2)	0.034 (3)	−0.0089 (18)	0.017 (2)	−0.002 (2)
C13'	0.038 (2)	0.046 (2)	0.040 (3)	0.006 (2)	0.020 (2)	0.007 (2)
C4	0.036 (2)	0.045 (2)	0.051 (3)	0.0009 (19)	0.011 (2)	−0.003 (2)
C13	0.034 (2)	0.041 (2)	0.040 (3)	−0.0028 (19)	0.002 (2)	−0.001 (2)
C18'	0.047 (3)	0.080 (3)	0.068 (4)	0.007 (2)	0.024 (3)	0.009 (3)
C11'	0.041 (2)	0.047 (3)	0.064 (4)	0.015 (2)	0.014 (3)	0.002 (3)
C17'	0.029 (2)	0.041 (2)	0.047 (3)	0.0018 (18)	0.004 (2)	−0.004 (2)
C11	0.037 (3)	0.044 (3)	0.094 (5)	−0.012 (2)	0.001 (3)	−0.008 (3)
C16	0.099 (4)	0.040 (3)	0.097 (5)	0.016 (3)	−0.046 (3)	0.004 (3)
C15	0.080 (4)	0.042 (3)	0.140 (6)	0.007 (3)	−0.016 (4)	−0.027 (3)
C6'	0.126 (4)	0.037 (3)	0.165 (7)	0.016 (3)	0.106 (5)	0.019 (3)
C19	0.056 (3)	0.040 (2)	0.066 (4)	−0.002 (2)	0.028 (3)	0.012 (2)
C15'	0.058 (3)	0.067 (3)	0.049 (3)	−0.013 (2)	0.015 (3)	−0.020 (3)
C19'	0.056 (3)	0.061 (3)	0.080 (4)	0.006 (2)	−0.001 (3)	0.018 (3)
C16'	0.079 (3)	0.029 (2)	0.080 (4)	0.002 (2)	0.033 (3)	0.004 (3)
C12	0.040 (2)	0.056 (3)	0.057 (4)	−0.013 (2)	0.008 (3)	0.011 (3)
C10	0.056 (3)	0.067 (3)	0.055 (4)	−0.012 (3)	0.009 (3)	−0.020 (3)
C4'	0.052 (3)	0.038 (2)	0.078 (4)	0.010 (2)	−0.003 (3)	−0.001 (3)
C18	0.057 (3)	0.061 (3)	0.086 (4)	0.003 (2)	0.051 (3)	0.008 (3)

### Geometric parameters (Å, °)

**Table:**

P1—O2	1.467 (2)	C5—H5B	0.9700
P1—O4	1.558 (2)	N2'—H2'B	0.8600
P1—O3	1.563 (2)	N2'—H2'C	0.8600
P1—C7	1.807 (3)	C5'—C6'	1.510 (5)
P1'—O2'	1.469 (2)	C5'—H5'A	0.9700
P1'—O4'	1.559 (2)	C5'—H5'B	0.9700
P1'—O3'	1.568 (2)	C10'—C11'	1.362 (5)
P1'—C7'	1.810 (3)	C10'—H10A	0.9300
O3—C14	1.443 (4)	C6—H6A	0.9600
O3'—C14'	1.461 (4)	C6—H6B	0.9600
N1—C1	1.356 (4)	C6—H6C	0.9600
N1—C7	1.458 (4)	C17—C19	1.496 (4)
N1—H1A	0.8600	C17—C18	1.503 (5)
O4—C17	1.463 (4)	C17—H17A	0.9800
O1—C1	1.220 (4)	C13'—H13A	0.9300
O4'—C17'	1.478 (4)	C4—H4A	0.9600
N1'—C1'	1.346 (4)	C4—H4B	0.9600
N1'—C7'	1.451 (4)	C4—H4C	0.9600
N1'—H1'A	0.8600	C13—C12	1.390 (5)
C7'—C8'	1.524 (4)	C13—H13B	0.9300
C7'—H7'A	0.9800	C18'—C17'	1.480 (6)
C1'—O1'	1.227 (4)	C18'—H18A	0.9600
C1'—C2'	1.515 (5)	C18'—H18B	0.9600
C7—C8	1.524 (4)	C18'—H18C	0.9600
C7—H7A	0.9800	C11'—H11A	0.9300
C8'—C13'	1.366 (5)	C17'—C19'	1.500 (5)
C8'—C9'	1.390 (5)	C17'—H17B	0.9800
C14'—C15'	1.475 (5)	C11—C10	1.355 (7)
C14'—C16'	1.512 (5)	C11—C12	1.369 (7)
C14'—H14A	0.9800	C11—H11B	0.9300
C8—C9	1.374 (5)	C16—H16A	0.9600
C8—C13	1.384 (5)	C16—H16B	0.9600
C2—N2	1.452 (4)	C16—H16C	0.9600
C2—C3	1.516 (5)	C15—H15A	0.9600
C2—C1	1.523 (5)	C15—H15B	0.9600
C2—H2A	0.9800	C15—H15C	0.9600
C3—C5	1.511 (4)	C6'—H6'A	0.9600
C3—C4	1.530 (5)	C6'—H6'B	0.9600
C3—H3A	0.9800	C6'—H6'C	0.9600
C3'—C4'	1.502 (5)	C19—H19A	0.9600
C3'—C5'	1.506 (5)	C19—H19B	0.9600
C3'—C2'	1.536 (5)	C19—H19C	0.9600
C3'—H3'A	0.9800	C15'—H15D	0.9600
C9'—C10'	1.367 (4)	C15'—H15E	0.9600
C9'—H9'A	0.9300	C15'—H15F	0.9600
C2'—N2'	1.450 (4)	C19'—H19D	0.9600
C2'—H2'A	0.9800	C19'—H19E	0.9600
C14—C16	1.464 (5)	C19'—H19F	0.9600
C14—C15	1.496 (6)	C16'—H16D	0.9600
C14—H14B	0.9800	C16'—H16E	0.9600
C9—C10	1.376 (5)	C16'—H16F	0.9600
C9—H9A	0.9300	C12—H12B	0.9300
C12'—C11'	1.366 (6)	C10—H10B	0.9300
C12'—C13'	1.399 (5)	C4'—H4'A	0.9600
C12'—H12A	0.9300	C4'—H4'B	0.9600
N2—H2B	0.8600	C4'—H4'C	0.9600
N2—H2C	0.8600	C18—H18D	0.9600
C5—C6	1.537 (5)	C18—H18E	0.9600
C5—H5A	0.9700	C18—H18F	0.9600
			
O2—P1—O4	110.01 (14)	C6'—C5'—H5'B	108.5
O2—P1—O3	114.41 (14)	H5'A—C5'—H5'B	107.5
O4—P1—O3	105.78 (12)	C11'—C10'—C9'	120.4 (4)
O2—P1—C7	113.18 (14)	C11'—C10'—H10A	119.8
O4—P1—C7	107.63 (14)	C9'—C10'—H10A	119.8
O3—P1—C7	105.33 (14)	C5—C6—H6A	109.5
O2'—P1'—O4'	116.83 (13)	C5—C6—H6B	109.5
O2'—P1'—O3'	113.99 (13)	H6A—C6—H6B	109.5
O4'—P1'—O3'	103.00 (13)	C5—C6—H6C	109.5
O2'—P1'—C7'	114.64 (16)	H6A—C6—H6C	109.5
O4'—P1'—C7'	101.38 (14)	H6B—C6—H6C	109.5
O3'—P1'—C7'	105.38 (14)	O4—C17—C19	106.7 (3)
C14—O3—P1	124.4 (2)	O4—C17—C18	108.7 (3)
C14'—O3'—P1'	124.4 (2)	C19—C17—C18	112.7 (3)
C1—N1—C7	121.7 (3)	O4—C17—H17A	109.6
C1—N1—H1A	119.2	C19—C17—H17A	109.6
C7—N1—H1A	119.2	C18—C17—H17A	109.6
C17—O4—P1	126.4 (2)	C8'—C13'—C12'	119.8 (4)
C17'—O4'—P1'	120.4 (2)	C8'—C13'—H13A	120.1
C1'—N1'—C7'	121.6 (3)	C12'—C13'—H13A	120.1
C1'—N1'—H1'A	119.2	C3—C4—H4A	109.5
C7'—N1'—H1'A	119.2	C3—C4—H4B	109.5
N1'—C7'—C8'	111.9 (3)	H4A—C4—H4B	109.5
N1'—C7'—P1'	110.6 (2)	C3—C4—H4C	109.5
C8'—C7'—P1'	110.8 (2)	H4A—C4—H4C	109.5
N1'—C7'—H7'A	107.8	H4B—C4—H4C	109.5
C8'—C7'—H7'A	107.8	C8—C13—C12	119.8 (4)
P1'—C7'—H7'A	107.8	C8—C13—H13B	120.1
O1'—C1'—N1'	122.3 (3)	C12—C13—H13B	120.1
O1'—C1'—C2'	120.4 (4)	C17'—C18'—H18A	109.5
N1'—C1'—C2'	117.3 (3)	C17'—C18'—H18B	109.5
N1—C7—C8	111.7 (3)	H18A—C18'—H18B	109.5
N1—C7—P1	111.0 (2)	C17'—C18'—H18C	109.5
C8—C7—P1	111.9 (2)	H18A—C18'—H18C	109.5
N1—C7—H7A	107.3	H18B—C18'—H18C	109.5
C8—C7—H7A	107.3	C10'—C11'—C12'	119.9 (4)
P1—C7—H7A	107.3	C10'—C11'—H11A	120.1
C13'—C8'—C9'	118.9 (3)	C12'—C11'—H11A	120.1
C13'—C8'—C7'	119.6 (3)	O4'—C17'—C18'	108.1 (3)
C9'—C8'—C7'	121.4 (3)	O4'—C17'—C19'	108.2 (3)
O3'—C14'—C15'	109.6 (3)	C18'—C17'—C19'	110.7 (3)
O3'—C14'—C16'	105.2 (3)	O4'—C17'—H17B	109.9
C15'—C14'—C16'	112.7 (3)	C18'—C17'—H17B	109.9
O3'—C14'—H14A	109.7	C19'—C17'—H17B	109.9
C15'—C14'—H14A	109.7	C10—C11—C12	119.9 (4)
C16'—C14'—H14A	109.7	C10—C11—H11B	120.0
C9—C8—C13	119.3 (3)	C12—C11—H11B	120.0
C9—C8—C7	120.4 (4)	C14—C16—H16A	109.5
C13—C8—C7	120.2 (4)	C14—C16—H16B	109.5
N2—C2—C3	111.1 (3)	H16A—C16—H16B	109.5
N2—C2—C1	110.8 (3)	C14—C16—H16C	109.5
C3—C2—C1	111.2 (3)	H16A—C16—H16C	109.5
N2—C2—H2A	107.9	H16B—C16—H16C	109.5
C3—C2—H2A	107.9	C14—C15—H15A	109.5
C1—C2—H2A	107.9	C14—C15—H15B	109.5
C5—C3—C2	111.6 (3)	H15A—C15—H15B	109.5
C5—C3—C4	111.5 (3)	C14—C15—H15C	109.5
C2—C3—C4	110.3 (3)	H15A—C15—H15C	109.5
C5—C3—H3A	107.7	H15B—C15—H15C	109.5
C2—C3—H3A	107.7	C5'—C6'—H6'A	109.5
C4—C3—H3A	107.7	C5'—C6'—H6'B	109.5
C4'—C3'—C5'	111.3 (4)	H6'A—C6'—H6'B	109.5
C4'—C3'—C2'	112.1 (3)	C5'—C6'—H6'C	109.5
C5'—C3'—C2'	111.4 (3)	H6'A—C6'—H6'C	109.5
C4'—C3'—H3'A	107.2	H6'B—C6'—H6'C	109.5
C5'—C3'—H3'A	107.2	C17—C19—H19A	109.5
C2'—C3'—H3'A	107.2	C17—C19—H19B	109.5
O1—C1—N1	123.1 (3)	H19A—C19—H19B	109.5
O1—C1—C2	121.9 (3)	C17—C19—H19C	109.5
N1—C1—C2	115.0 (3)	H19A—C19—H19C	109.5
C10'—C9'—C8'	120.7 (4)	H19B—C19—H19C	109.5
C10'—C9'—H9'A	119.7	C14'—C15'—H15D	109.5
C8'—C9'—H9'A	119.7	C14'—C15'—H15E	109.5
N2'—C2'—C1'	110.0 (3)	H15D—C15'—H15E	109.5
N2'—C2'—C3'	110.8 (3)	C14'—C15'—H15F	109.5
C1'—C2'—C3'	110.2 (3)	H15D—C15'—H15F	109.5
N2'—C2'—H2'A	108.6	H15E—C15'—H15F	109.5
C1'—C2'—H2'A	108.6	C17'—C19'—H19D	109.5
C3'—C2'—H2'A	108.6	C17'—C19'—H19E	109.5
O3—C14—C16	109.5 (3)	H19D—C19'—H19E	109.5
O3—C14—C15	109.0 (4)	C17'—C19'—H19F	109.5
C16—C14—C15	110.6 (3)	H19D—C19'—H19F	109.5
O3—C14—H14B	109.2	H19E—C19'—H19F	109.5
C16—C14—H14B	109.2	C14'—C16'—H16D	109.5
C15—C14—H14B	109.2	C14'—C16'—H16E	109.5
C8—C9—C10	119.9 (4)	H16D—C16'—H16E	109.5
C8—C9—H9A	120.1	C14'—C16'—H16F	109.5
C10—C9—H9A	120.1	H16D—C16'—H16F	109.5
C11'—C12'—C13'	120.3 (4)	H16E—C16'—H16F	109.5
C11'—C12'—H12A	119.9	C11—C12—C13	119.8 (5)
C13'—C12'—H12A	119.9	C11—C12—H12B	120.1
C2—N2—H2B	120.0	C13—C12—H12B	120.1
C2—N2—H2C	120.0	C11—C10—C9	121.1 (5)
H2B—N2—H2C	120.0	C11—C10—H10B	119.5
C3—C5—C6	112.9 (3)	C9—C10—H10B	119.5
C3—C5—H5A	109.0	C3'—C4'—H4'A	109.5
C6—C5—H5A	109.0	C3'—C4'—H4'B	109.5
C3—C5—H5B	109.0	H4'A—C4'—H4'B	109.5
C6—C5—H5B	109.0	C3'—C4'—H4'C	109.5
H5A—C5—H5B	107.8	H4'A—C4'—H4'C	109.5
C2'—N2'—H2'B	120.0	H4'B—C4'—H4'C	109.5
C2'—N2'—H2'C	120.0	C17—C18—H18D	109.5
H2'B—N2'—H2'C	120.0	C17—C18—H18E	109.5
C3'—C5'—C6'	115.0 (3)	H18D—C18—H18E	109.5
C3'—C5'—H5'A	108.5	C17—C18—H18F	109.5
C6'—C5'—H5'A	108.5	H18D—C18—H18F	109.5
C3'—C5'—H5'B	108.5	H18E—C18—H18F	109.5
			
O2—P1—O3—C14	20.4 (3)	N2—C2—C3—C4	173.5 (3)
O4—P1—O3—C14	141.7 (3)	C1—C2—C3—C4	49.6 (4)
C7—P1—O3—C14	−104.5 (3)	C7—N1—C1—O1	−6.1 (5)
O2'—P1'—O3'—C14'	−29.8 (3)	C7—N1—C1—C2	175.4 (3)
O4'—P1'—O3'—C14'	−157.4 (3)	N2—C2—C1—O1	−78.0 (5)
C7'—P1'—O3'—C14'	96.7 (3)	C3—C2—C1—O1	46.1 (5)
O2—P1—O4—C17	178.8 (2)	N2—C2—C1—N1	100.6 (4)
O3—P1—O4—C17	54.8 (3)	C3—C2—C1—N1	−135.3 (3)
C7—P1—O4—C17	−57.5 (3)	C13'—C8'—C9'—C10'	0.8 (5)
O2'—P1'—O4'—C17'	−56.4 (3)	C7'—C8'—C9'—C10'	179.5 (3)
O3'—P1'—O4'—C17'	69.4 (3)	O1'—C1'—C2'—N2'	−42.7 (5)
C7'—P1'—O4'—C17'	178.3 (3)	N1'—C1'—C2'—N2'	137.5 (3)
C1'—N1'—C7'—C8'	115.2 (3)	O1'—C1'—C2'—C3'	79.8 (4)
C1'—N1'—C7'—P1'	−120.7 (3)	N1'—C1'—C2'—C3'	−100.0 (4)
O2'—P1'—C7'—N1'	−60.2 (3)	C4'—C3'—C2'—N2'	176.9 (3)
O4'—P1'—C7'—N1'	66.6 (3)	C5'—C3'—C2'—N2'	−57.6 (5)
O3'—P1'—C7'—N1'	173.6 (2)	C4'—C3'—C2'—C1'	54.9 (5)
O2'—P1'—C7'—C8'	64.5 (3)	C5'—C3'—C2'—C1'	−179.6 (3)
O4'—P1'—C7'—C8'	−168.7 (2)	P1—O3—C14—C16	−109.8 (4)
O3'—P1'—C7'—C8'	−61.6 (3)	P1—O3—C14—C15	129.1 (3)
C7'—N1'—C1'—O1'	2.7 (5)	C13—C8—C9—C10	−1.3 (6)
C7'—N1'—C1'—C2'	−177.5 (3)	C7—C8—C9—C10	179.2 (3)
C1—N1—C7—C8	−125.8 (3)	C2—C3—C5—C6	162.1 (3)
C1—N1—C7—P1	108.6 (3)	C4—C3—C5—C6	−74.0 (4)
O2—P1—C7—N1	55.9 (3)	C4'—C3'—C5'—C6'	−58.0 (5)
O4—P1—C7—N1	−65.9 (2)	C2'—C3'—C5'—C6'	176.1 (4)
O3—P1—C7—N1	−178.4 (2)	C8'—C9'—C10'—C11'	−1.0 (6)
O2—P1—C7—C8	−69.6 (3)	P1—O4—C17—C19	144.1 (2)
O4—P1—C7—C8	168.6 (2)	P1—O4—C17—C18	−94.2 (3)
O3—P1—C7—C8	56.1 (3)	C9'—C8'—C13'—C12'	−0.9 (5)
N1'—C7'—C8'—C13'	−133.4 (3)	C7'—C8'—C13'—C12'	−179.6 (3)
P1'—C7'—C8'—C13'	102.7 (3)	C11'—C12'—C13'—C8'	1.3 (6)
N1'—C7'—C8'—C9'	48.0 (4)	C9—C8—C13—C12	2.2 (5)
P1'—C7'—C8'—C9'	−76.0 (4)	C7—C8—C13—C12	−178.2 (3)
P1'—O3'—C14'—C15'	89.5 (3)	C9'—C10'—C11'—C12'	1.3 (6)
P1'—O3'—C14'—C16'	−149.1 (2)	C13'—C12'—C11'—C10'	−1.5 (7)
N1—C7—C8—C9	115.1 (4)	P1'—O4'—C17'—C18'	−121.1 (3)
P1—C7—C8—C9	−119.8 (3)	P1'—O4'—C17'—C19'	118.9 (3)
N1—C7—C8—C13	−64.4 (4)	C10—C11—C12—C13	−1.4 (7)
P1—C7—C8—C13	60.7 (4)	C8—C13—C12—C11	−0.9 (6)
N2—C2—C3—C5	−61.9 (4)	C12—C11—C10—C9	2.4 (7)
C1—C2—C3—C5	174.2 (3)	C8—C9—C10—C11	−1.0 (6)

### Hydrogen-bond geometry (Å, °)

**Table:**

*D*—H···*A*	*D*—H	H···*A*	*D*···*A*	*D*—H···*A*
N1—H1A···O2'^i^	0.86	2.07	2.913 (4)	166
N1'—H1'A···O2^ii^	0.86	1.98	2.833 (4)	171

Symmetry codes: (i) −*x*+1, *y*−1/2, −*z*+1; (ii) −*x*+1, *y*+1/2, −*z*+1.
